# Analysis of cell proliferation and tissue remodelling uncovers a KLF4 activity score associated with poor prognosis in colorectal cancer

**DOI:** 10.1038/s41416-018-0253-0

**Published:** 2018-10-05

**Authors:** Silvia Halim, Elke K. Markert, Alexei Vazquez

**Affiliations:** 1 Cancer Research UK Beatson Institute, Glasgow, UK; 2 Institute of Cancer Sciences, University of Glasgow, Glasgow, UK

## Abstract

**Background:**

Human cancers can be classified based on gene signatures quantifying the degree of cell proliferation and tissue remodelling (PR). However, the specific factors that drive the increased tissue remodelling in tumours are not fully understood. Here we address this question using colorectal cancer as a case study.

**Methods:**

We reanalysed a reported cohort of colorectal cancer patients. The patients were stratified based on gene signatures of cell proliferation and tissue remodelling. Putative transcription factors activity was inferred using gene expression profiles and annotations of transcription factor targets as input.

**Results:**

We demonstrate that the PR classification performs better than the currently adopted consensus molecular subtyping (CMS). Although CMS classification differentiates patients with a mesenchymal signature, it cannot distinguish the remaining patients based on survival. We demonstrate that the missing factor is cell proliferation, which is indicative of good prognosis. We also uncover a KLF4 transcription factor activity score associated with the tissue remodelling gene signature. We further show that the KLF4 activity score is significantly higher in colorectal tumours with predicted infiltration of cells from the myeloid lineage.

**Conclusion:**

The KLF4 activity score is associated with tissue remodelling, myeloid cell infiltration and poor prognosis in colorectal cancer.

## Background

There have been several attempts to classify colorectal cancer patients into subtypes based on the analysis of gene expression signatures and prognosis. Anjomshoaa et al.^1^ developed a colon-specific gene proliferation signature and reported that patients with a low proliferative signature had shorter disease-free survival. Loboda et al.^2^ reported that a signature of epithelial-mesenchymal transition (EMT) was predictive of poor outcome in colorectal cancer. Later on, Markert et al.^3^ unified these two previous approaches and demonstrated that human cancers, including colorectal cancer, can be classified based on gene expression signatures quantifying the degree of cell proliferation and tissue remodelling (PR). More recently, a colorectal cancer subtyping consortium (CRCSC) adopted an unsupervised clustering approach to stratify colorectal cancers based on their gene expression profiles.^4^ This consensus method resulted in a classification of colorectal cancer into four subtypes: CMS1, CMS2, CMS3 and CMS4, where CMS stands for consensus molecular subtypes. The CMS4 subtype was enriched for gene signatures of EMT, indicating that this subtype is characterized by a high degree of tissue remodelling.

These studies unanimously identified gene signatures of EMT or tissue remodelling as a major indicator of poor prognosis in colorectal cancer. Yet, the cell proliferation gene signature is missing in the CMS scheme and it is not clear how this affects the CMS ability to stratify colorectal cancer patients beyond the EMT subtype (CMS4). More importantly, the molecular pathways driving tissue remodelling in colorectal cancer are not fully understood. Here we address these two issues using a systems biology approach. First, we present a side-by-side comparison of the CMS and PR classifications of colorectal cancer. We show that the cell proliferation gene signature can significantly differentiate patients of the EMT subtype based on survival. Second, we identify transcription factor (TF) activity scores that correlate with the PR gene signatures. Among them, we follow up on KLF4 activity that we predict to be a driver of tissue remodelling in colorectal cancer. We validate the KLF4 activity score by showing its increased expression in immune cells of the myeloid lineage, which are known to be regulated by KLF4.^5^ We further show that, in colorectal cancer samples, the KLF4 activity score is associated with myeloid cell infiltration. These findings indicate that the TF KLF4 is associated with tissue remodelling in colorectal cancer via myeloid cell infiltration.

## Methods

### CRCSC gene expression data

Normalised gene expression datasets of colorectal cancer tumour samples were obtained from Synapse (Synapse ID syn2634742). The data hosted under this Synapse ID consists of datasets from Gene Expression Omnibus (GEO): GSE13067, GSE13294, GSE14333, GSE17536, GSE20916, GSE2109, GSE23878, GSE33113, GSE37892 and GSE39582; and The Cancer Genome Atlas (TCGA). Gene expression data normalisation, outlier sample detection and other pre-processing steps can be found in the original research article.^4^ For each GEO dataset, only probes that have gene annotation were included for subsequent analyses. The most variable probe (a probe with largest interquartile range) was then selected for each gene. Subsequently, all GEO and TCGA gene expression datasets were corrected for mean-centering for each gene. For a given gene, mean-centering was performed by subtracting the expression values of all samples from the mean expression value across all samples and it was performed for all genes in the dataset.

### Human immune cell types gene expression data

Human immune cell transcriptome data with accession number GSE3982 was downloaded from GEO. The gene expression data were quantile-normalised based on ‘preprocessCore’ bioconductor package and then log2 transformed. Only probes that had gene annotation were included for subsequent analyses. The most variable probe (a probe with largest interquartile range) was then selected for each gene. Subsequently, the expression data were mean-centered for each gene.

### Gene signatures of PR

Gene signatures for cell proliferation (P) and tissue remodelling (R) were obtained from ref. ^3^

### Gene set enrichment analysis (GSEA)

Given the gene expression values of *n* genes across tumour samples and a gene set *L* containing *m* genes as input, we estimated the enrichment of *L* genes within the tail of low or high expression values using GSEA.^6^ To this end, we determined the sample-dependent rank vector *g*_*ik*_, denoting the *i*-th gene with largest expression value in the sample *k*. Then the running enrichment score $E_{ik} = \mathop {\sum}\limits_{j = 1}^i {h_{g_{jk}}}$ was calculated, where *h*_*g*_ = 1/*m* if *g* ∈ *L* and *h*_*g*_ = −1/(*n*−*m*) otherwise. The enrichment of *L* genes within the tail of low and high expression values is quantified by $E_{k - } = \mathop {{\min}}\limits_i E_{ik}$ and $E_{k + } = \mathop {{\max}}\limits_i E_{ik}$, respectively.^6^ A permutation test was used as a non-parametric estimate of the statistical significance of the signature scores. Specifically, *n*_*P*_ = 100,000 permutations of the gene expression values were generated and their corresponding signature scores *E*_*l*_ (*l* = 1,…,*n*_*P*_) were calculated. The least bias estimate of the statistical significance of *E*_*k*__−_ and *E*_*k*+_ being high are $p_{k - } = \left( {1 + \Sigma _{l|E_l \le E_{k - }}1} \right)/\left( {1 + n_P} \right)$ and $p_{k + } = \left( {1 + \Sigma _{l|E_l \ge E_{k + }}1} \right)/\left( {1 + n_P} \right)$.^7^ Finally, when *p*_*k*−_ < *p*_*k*+_ we report the signature score *E*_*k*_ = *E*_*k*__−_ or *E*_*k*_ = *E*_*k*+_ otherwise.

### Inference of TF activity scores

The TF activity scores were inferred using the linear least squares model, $G_{ik} = \mathop {\sum}\nolimits_j {T_{ij}A_{jk}}$ where *A*_*jk*_ is the activity of TF *j* in sample *k*, *G*_*ik*_ is the expression of gene *i* in sample *k*, and *T*_*ij*_ = −1,0,1 if TF *j* negatively regulates, does not regulate or positively regulates gene *i*, respectively. The matrix *T* was constructed using the Transcriptional Regulatory Relationships Unravelled by Sentence-based Text-mining (TTRUST) database^8^ as input. At the time of download, this database contained annotations for 748 human TFs, 2374 unique target genes and 8015 transcriptional regulatory relationships. It is a manually curated database with experimentally validated interactions and it provides information on the mode of regulation, i.e., activating or repressing. The database contains unknown interactions but only activating and repressing interactions were included for analysis. Using the gene expression matrix *G* reported for the colorectal tumour samples and the transcription regulation matrix *T* derived from the TTRUST database as input, we inferred the TF activity matrix *A*. The inference was carried out by solving the system of linear equations reported above in the least squares sense, using the R function *lsfit*. The R function to perform the TF activity estimation is provided in the Supplementary information, inferTFactivityScores.r (Dataset 4). Only TFs with standard deviation of activity scores across tumour samples not equal to zero were retained for further analyses. A permutation test was used as a non-parametric estimate of the statistical significance of the observed activity score *x*_*j*0_ of TF *j* being high on a given sample of the CRCSC cohort. To this end we generated 100 permutations of each of the 2423 gene expression samples in the CRCSC cohort and inferred the TF activity scores for these permuted samples, resulting in *x*_*jk*_ (*k* = 1,…,*n* = 242,300) reference scores for the activity of each TF *j*. The least bias estimate of the statistical significance of *s*_*j*0_ being high is $p_j = ( {1 + \mathop {\sum}\nolimits_{S_{jk} \ge S_{j0}} 1 })/\left( {1 + n} \right)$.^7^ A gene expression sample was called positive for high TF *j* activity score if *p*_*j*_ < 0.05, and negative otherwise.

### Multiple testing correction

When performing multiple testing, the statistical significance was corrected following Benjamini-Hochberg (BH) procedure to control the false discovery rate (FDR).

### Estimation of immune cell types abundance from gene expression data

All gene expression datasets from the CRCSC were combined. The composition of immune cell types in all of the samples was estimated using CIBERSORT.^9^ For running ‘CIBERSORT’ function, LM22 signature genes file provided by CIBERSORT was used as ‘sig_matrix’ variable and the combined gene expression data was used as the mixture file. The function was run with 1000 permutations and quantile normalisation. The resulted compositions of each immune cell for all samples were then correlated with KLF4 activity of all samples using Spearman’s rank correlation coefficient. To identify which immune cells that are significantly correlated with the level of KLF4 activity, *p*-values were calculated for the correlations following the least bias estimate of the statistical significance. To assess the abundance of immune cells in each PK subtypes, all samples that belong to a PK subtype were grouped together and the compositions of the immune cell of interest in these samples were then visualised.

### Univariate survival analysis

Cox proportional hazards regression model was fitted for survival analysis. It was performed using overall survival or relapse-free survival information and PR or PK or CMS sample membership for analysis based on PR or PK or CMS classification, respectively. *P*-value from log-rank test was reported as the significance of the classification in predicting an event occurring, i.e., death or relapse in overall survival or relapse-free survival data, respectively. *P*-value < 0.05 was reported as a significant result in all cases.

### Multivariate survival analysis

Cox proportional hazards regression model was fitted for survival analysis and it was performed using overall survival or relapse-free survival information, stage, age, gender, and P and R enrichment scores. *P*-value from Wald test was reported as the significance of each covariate (P or R enrichment scores, stage, age or gender) in predicting an event occurring, i.e., death or relapse, while taking into account all other covariates. *P*-value < 0.05 was reported as a significant result in all cases.

## Results

### CMS versus PR classifications of colorectal cancer

**Fig. 1:**
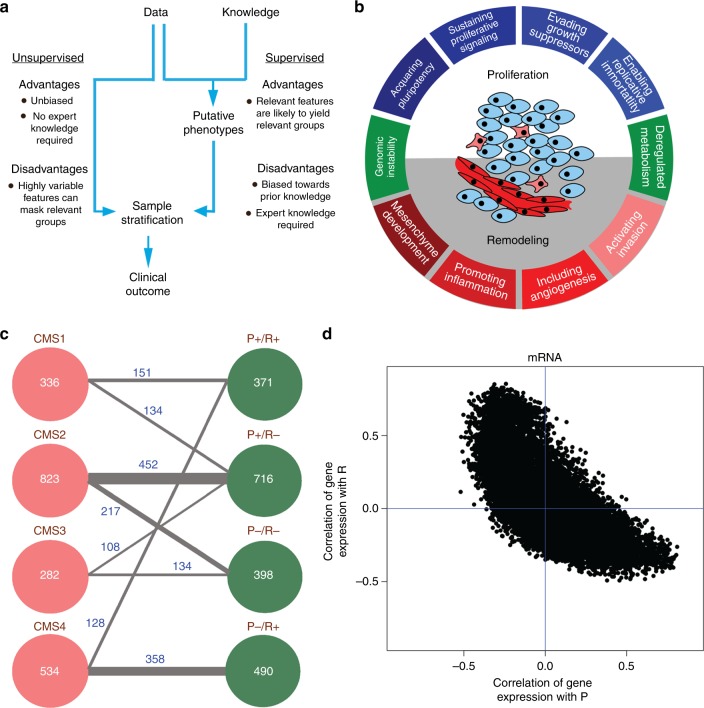
CMS vs PR classifications of colorectal cancer. **a** Flow diagrams of the unsupervised clustering approach and the reductionist supervised approach to patient stratification. **b** The hallmarks of cancer drive two major features of cancer: cell proliferation and tissue remodelling. **c** Mapping between the CMS and PR subtypes. The lines connecting the circles show the largest and second largest overlaps among subtypes from both approaches. **d** Scatter plot of the Spearman’s rank correlation between gene expression and the tissue remodelling signature (*Y*-axis) as a function of the Spearman’s rank correlation between the gene expression and the cell proliferation signature (*X*-axis). Each symbol represents a gene and the Spearman’s rank correlations were calculated across colorectal cancer patients

We started with a side-by-side comparison of the performance of the colorectal cancer unsupervised and supervised classifications (Fig. 1a). The unsupervised classification is represented by CMS. The CMS scheme does not use previous biological knowledge as input and, in this sense it is not biased or supervised. The supervised classification is represented by the PR scheme. The PR scheme is based on our observation that the hallmarks of cancer can be conceptually arranged into two groups representing processes that promote proliferation or tissue remodelling (Fig. 1b, ref. ^3^). In this sense, it is biased and fully based on previous biological knowledge.

We performed a meta-analysis of the same cohort of colorectal cancer patients used to develop the CMS scheme.^4^ This cohort brings together gene expression and survival data reported in multiple studies. These samples had been previously stratified according to the CMS scheme, resulting in four subtypes, namely CMS1,2,3,4.^4^ We reclassified all patients using our PR approach (Fig. 1b). To this end, we quantified the degree of PR in each patient sample. When the cell proliferation signature was significantly up-regulated, the sample was classified as P+, and P− if otherwise. When the tissue remodelling signature was significantly up-regulated, the sample was classified as R+, and R− if otherwise. By construction, this classification results in four subtypes, namely P−/R−, P−/R+, P+/R− and P+/R+. The assignment of each patient to the PR subtypes is reported in the Supporting Information, Dataset 1.

Although these two classifications were carried out independently, the resulting subtypes manifest some overlap (Fig. 1c). The CMS2 subtype maps to a great extent to the P+/R− subtype (statistical significance *p* = 2.3 × 10^−^^48^, one-tailed Fisher’s exact test) and the CMS4 subtype maps to a great extent to the P−/R+ subtype (statistical significance *p* = 8.9 × 10^−^^144^, one-tailed Fisher’s exact test). This overlap between the unbiased CMS classification and the PR classification can be explained by the strong correlation between several genes and the gene signature of tissue remodelling (Fig. 1d). A high percentage of the expressed genome is significantly correlated with the tissue remodelling enrichment score (34.25%, *p* < 0.05 in permutation test for Spearman correlation). Therefore, it is expected that any unbiased clustering method will reflect this strong signal. To analyse this whole genome biases more systematically, we calculated the Spearman correlation coefficients *S*(G,P) and *S*(G,R), quantifying the correlation between the expression of a given gene G and the P and R enrichment scores across the tumour samples. We observed that *S*(G,P) and *S*(G,R) are significantly and negatively correlated across genes (Fig. 1d, *S* = −0.70, *p* = 1 × 10^−^^5^, permutation test). In other words, there is a large group of genes whose expression is highly and positively correlated with the R enrichment score, but negatively correlated with the P enrichment score. Vice versa, there is a large group of genes whose expression is highly and positively correlated with the P enrichment score, but negatively correlated with the R enrichment score.

**Fig. 2:**
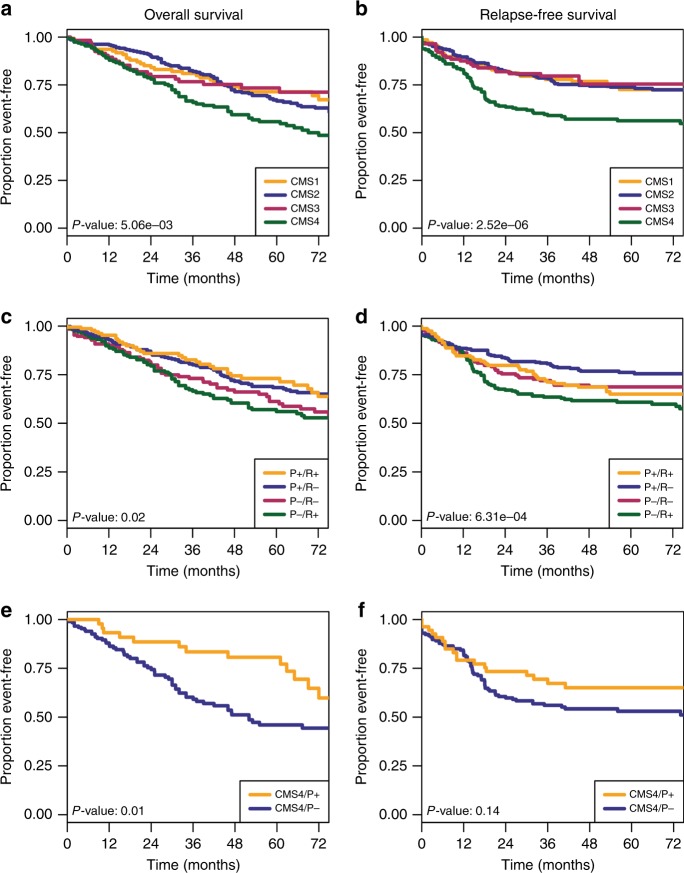
Survival analysis based on the CMS or PR classifications. Kaplan–Meier plots of colorectal cancer subtypes survival, based on the CMS and PR classification schemes. *P*-values report statistical significance based on log-rank test. **a** CMS stratification, overall survival. **b** CMS stratification, relapse-free survival. **c** PR stratification, overall survival. **d** PR stratification, relapse-free survival. **e**, **f** Overall survival (**e**) and relapse-free survival (**f**) of CMS4 patients stratified by the P signature

Next, we compared the performance of the CMS and PR approaches in stratifying patients based on overall and relapse-free survival (Fig. 2a–d). In terms of splitting of the survival curves, both approaches achieve statistical significance for these outcomes. The CMS4 subtype in the unsupervised scheme and the corresponding P−/R+ group in the supervised scheme exhibit the worst prognosis. However, the unsupervised approach cannot distinguish the rest of the patients based on survival. In other words, the CMS classification differentiates the patients with tissue remodelling but it fails to distinguish the remaining patients based on survival. In contrast, the two features of the PR classification associate with outcome. In addition to distinguishing the tissue remodelling group (P−/R+), it shows that the P+/R− subtype exhibits significantly better prognosis than the P−/R− group. The relevance of the cell proliferation gene signature is further demonstrated when we split the CMS4 group into patients with significant cell proliferation gene signature (CMS4/P+) and the remaining (CMS4/P−). The patients in the CMS4/P+ exhibit a significantly better overall survival (Fig. 2e, *p* = 0.01, log-rank test) and a trend towards better relapse-free survival (Fig. 2f, *p* = 0.14, log-rank test) than the CMS4/P− group. Therefore, the PR classification correctly highlights the additional observation that cell proliferation is indicative of good prognosis in the context of colorectal cancer, as a second prognostic factor besides a tissue remodelling signature.

Finally, we conducted a multivariate analysis to determine whether the P and R enrichment scores are independent prognostic factors after correcting for clinical variables. The information about whether patients received treatment and what type of treatment was given was not reported and, therefore, we could not include treatment options as a variable in the multivariate analysis. Yet, age and stage were reported, two clinical variables that are often used to make treatment decisions. In summary, the multivariate analysis includes the P and R enrichment scores together with the clinical variables age, stage and gender. As expected, stage is significantly associated with an increased risk of death and relapse (Table 1a). The P enrichment score exhibits a significant association with reduced risk of death, while the R enrichment score exhibits a significant association with increased risk of relapse (Table 1a). When we excluded the R enrichment score, the P enrichment score exhibited a significant association with reduced risk of both death and relapse (Table 1b). Similarly, when we excluded the P enrichment score, then the R enrichment score exhibited a significant association with increased risk of both death and relapse. This analysis indicates that the P and R enrichment scores are not independent as prognostic factors when considered as real value variables (as oppose to categorical +/−). Indeed, the P and R enrichment scores are strongly negatively correlated (*S* = −0.44, *p* = 1 × 10^−^^5^, permutation test).

**Table 1:** Multivariate survival analysis

	Overall survival			Relapse-free survival		
Variable	Hazard ratio	95% Confidence interval	*P*-value	Hazard ratio	95% Confidence interval	*P*-value
(a) P & R						
P scores	0.32	0.1028–0.988	0.05	0.41	0.1322–1.260	0.12
R scores	1.32	0.3697–4.703	0.67	4.60	1.1443–18.516	0.03
Stage	1.98	1.6686–2.338	2.6E-15	2.86	2.3485–3.488	<2e-16
Age	1.03	1.0209–1.042	4.6E-09	1.00	0.9931–1.013	0.56
Gender	1.26	0.9854–1.612	0.07	1.36	1.0488–1.773	0.02
(b) P						
P scores	0.28	0.1052–0.7598	1.2E-02	0.23	0.08452–0.6208	3.8E-03
Stage	1.98	1.6708–2.3399	2.1E-15	2.87	2.35931–3.4884	<2e-16
Age	1.03	1.0209–1.0423	4.8E-09	1.00	0.99296–1.0126	0.58
Gender	1.26	0.9823–1.6047	0.07	1.34	1.03201–1.7422	0.03
(c) R						
R scores	2.46	0.8085–7.454	1.1E-01	7.76	2.2706–26.494	1.1E-03
Stage	2.00	1.6887–2.365	7.8E-16	2.90	2.3834–3.539	<2e-16
Age	1.03	1.0202–1.041	8.4E-09	1.00	0.9926–1.012	0.63
Gender	1.28	0.9979–1.632	0.05	1.38	1.0621–1.794	0.02

Multivariate survival analysis considering the clinical variables stage, age and gender together with the enrichment scores for a) P and R, b) P only and c) R only

### Putative TFs driving the PR subtypes

The PR gene expression signatures could be driven by multiple factors. Cell proliferation could reflect an enrichment of epithelial cell types at expenses of depletion of stromal cell types. In turn, tissue remodelling could be the consequence of multiple processes such as wound healing or immune cell infiltration. To address the latter we determined the correlation between the R enrichment scores and the enrichment scores for multiple gene signatures associated with tissue remodelling (Supporting Information, Dataset 2). We found the R enrichment scores to be highly correlated with gene signatures for “Response to wounding” (GO:0009611, *S* = 0.95, *p* = 1 × 10^−^^5^), “Stromal tissue” (ref. ^10^, *S* = 0.90, *p* = 1 × 10^−^^5^), “Immune cell infiltration” (ref. ^10^, *S* = 0.74, *p* = 1 × 10^−^^5^), “Mesenchyme development” (GO:0060485, *S* = 0.72, *p* = 1 × 10^−^^5^) and “Epithelial Mensechymal Transition” (GO:0001837, *S* = 0.65, *p* = 1 × 10^−^^5^), where GO denotes a Gene Ontology gene set. From these associations we cannot determine which process or combinations of processes is driving tissue remodelling.

**Fig. 3:**
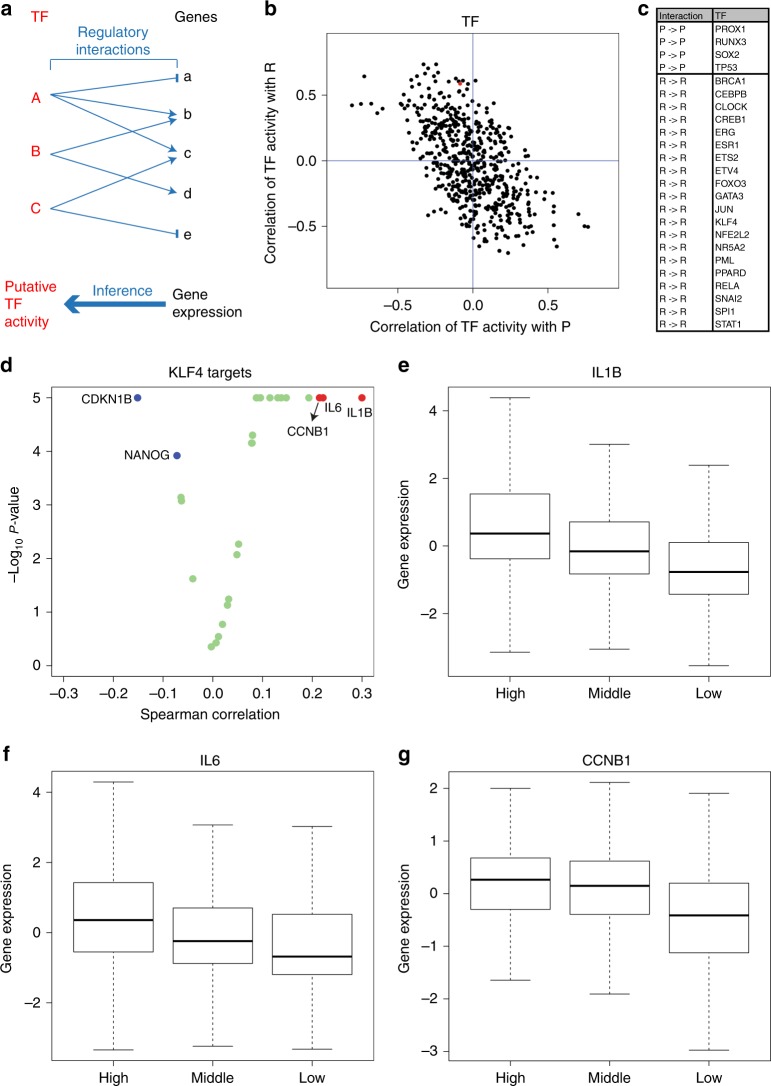
KLF4 activity score associated with tissue remodelling. **a** Diagram of gene regulation and transcription activity inference. **b** Scatter plot of the Spearman’s rank correlation between a transcription factor signature and the tissue remodelling signature (*Y*-axis) as a function of the Spearman’s rank correlation between the transcription factor signature and the cell proliferation signature (*X*-axis). Each symbol represents a transcription factor and the Spearman’s rank correlations were calculated across colorectal cancer patients. The transcription factor highlighted in red is KLF4. **c** List of putative transcription factors sustaining the transcriptional programmes of cell proliferation (P→P) or tissue remodelling (R→R). **d** Volcano plot of statistical significance vs Spearman’s rank correlation between the expression of annotated KLF4 targets and the KLF4 activity score. **e**–**g** Expression of selected KLF4 targets across patients with high, middle and low KLF4 activity score

TF activities control the maintenance of cell types and the transition between them. However, quantifying the activity of TFs in tumour samples is challenging. TFs are often regulated at the post-transcriptional level and, therefore, their gene expression is not sufficient to predict its activity. Measuring TF protein expression levels would be more accurate, but is not measured on a regular basis at the proteome-wide scale. To tackle this problem, we developed a linear regression method to infer TF activities. The method uses gene expression profiles and annotations of TF targets with their specific actions as input, i.e., activation or repression of target genes (Fig. 3a). The outcome is a putative transcriptional activity for every annotated TF, herein referred to as TF activity score. Using this approach, we inferred the TF activity score for each annotated TF on each patient in the same cohort of colorectal cancer patients.

First, we focused on associations between the inferred TF activities and the gene signatures for PR. To this end, for a transcription factor TF, we calculated the Spearman correlation coefficients *S*(TF,P) and *S*(TF,R) between the TF activity score and the P and R enrichment scores across the colorectal tumour samples. We observed that TFs manifesting high *S*(TF,P) exhibit a high but negative *S*(TF,R) and vice versa (Fig. 3b). There is indeed a strong negative correlation between *S*(TF,P) and *S*(TF,R) across TFs (Fig. 3b, *S* = −0.56, *p* = 1 × 10^−^^5^, permutation test). This indicates that PR biases in gene expression (Fig. 1d) are driven by biases in the transcriptional gene expression programmes.

Next, we aimed to uncover TFs whose putative activity is correlated with the cell proliferation or tissue remodelling enrichment scores and at the same time, the expression of their annotated targets is associated with these enrichment scores as well (Fig. 3c). This analysis can yield different patterns of regulation depending on the association of the TF activity scores and the P or R enrichment scores, the type of regulation of its target genes (activation/repression) and the association of the expression of target genes with the P or R gene signatures. From the biological point of view, we are more interested in patterns of regulation that aim to sustain one specific transcriptional programme. That includes the case where a TF activity score is correlated with the cell proliferation enrichment score and the expression of its target genes is also correlated with the cell proliferation enrichment score (P→P) and the equivalent relationship for tissue remodelling (R→R). Following this rationale, we identified the TFs PROX1, RUNX3, SOX2 and TP53 as candidates for sustaining the cell proliferation programme in colorectal cancer (Fig. 3c and Table S1, P→P). In turn, we identified the TFs BRCA1, CEBPB, CLOCK, CREB1, ERG, ESR1, ETS2, ETV4, FOXO3, GATA3, JUN, KLF4, NFE2L2, NR5A2, PML, PPARD, RELA, SNAI2, SPI1 and STAT1 as candidates for sustaining the tissue-remodelling programme (Fig. 3c and Table S1, R→R). The full list of TFs together with the association of their activity scores with the P and R enrichment scores is reported in the Supporting Information, Dataset 3.

### KLF4 activity score is associated with myeloid cell infiltration

From the analysis of TF activity scores we identified KLF4 as a putative TF promoting tissue remodelling. Specifically, the KLF4 activity score is significantly and positively correlated with the R enrichment score (*S* = 0.59, *p* = 1 × 10^−^^5^, permutation test) and the KLF4 targets are enriched for genes whose expression is significantly and positively correlated with the tissue remodelling signature (*S* = 0.18, *p* = 1 × 10^−^^5^, Gene set enrichment analysis (GSEA) test). KLF4 has been linked to pluripotency^11, 12^ and to myeloid subtypes of the immune system. Both of these features contribute to poor prognosis in colorectal cancer. We therefore decided to investigate this TF in further detail.

First, we took a closer look at the genes annotated as KLF4 targets. About 50% of the KLF4 targets exhibit a significant correlation between their expression and the KLF4 activity scores (Fig. 3d, Table S2), indicating that not all KLF4 targets contribute to the inferred KLF4 activity score. We note that discrepancies between activity score of a TF and the expression of one or more of its targets are expected, because TFs have multiple targets and genes can be regulated by multiple TFs. Furthermore, the genes whose expression is significantly correlated with the KLF4 activity score, exhibit a small correlation coefficient, indicating that no single KLF4 target can replace the other genes in deriving at the KLF4 activity score. This is as illustrated in Fig. 3e–g for the three most correlated genes. The average expression of these genes increases from patient groups having low to high KLF4 activity scores but the fluctuation within each group is high. In summary, the KLF4 activity score is an aggregate signal taking into account the concomitant expression of several KLF4 targets.

As mentioned above KLF4 has been reported for its role in myelopoiesis,^5^ suggesting the hypothesis that the tissue remodelling signature is in part associated with immune cell infiltration. In line with these observations, the two KLF4 targets with the highest correlation with the KLF4 activity score are the *IL1B* and *IL6* genes encoding for cytokines secreted by cells of the immune system (Fig. 3d). Based on this evidence we hypothesised that the KLF4 activity may derive from immune cells penetrating the tumour.

**Fig. 4:**
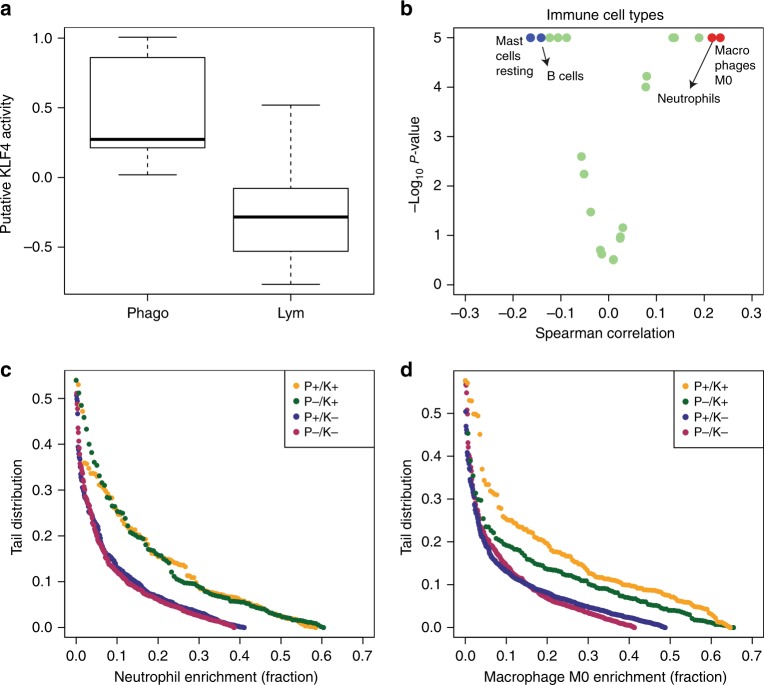
KLF4 activity score in colorectal cancer. **a** Expression of the KLF4 signature across phagocytes (myeloid) and lymphoid immune cell types cultured in vitro. **b** Volcano plot of statistical significance vs Spearman’s rank correlation between inferred immune cell fractions and KLF4 activity score in colorectal tumours. Each point represents an immune cell type. Annotated immune cell types from left to right in the plot are mast cells (resting), B-cells (naive), neutrophils and macrophages (M0). **c**, **d** Tail distribution of the enrichment of **c** neutrophils and **d** macrophages M0 across colorectal tumours divided according to their P and K status

To start addressing the relationship between KLF4 and the immune system, we first determined whether the TF activity score approach was valid in the context of pure immune cell populations. To this end, we used a reported transcriptome dataset quantifying genome-wide expression in sorted immune cell types (ref. ^13^, GEO accession code GSE3982). To this dataset we applied our TF activity inference approach, obtaining a quantification of the KLF4 activity score for each sample. The predicted KLF4 activity score was found to be significantly up-regulated in the phagocytes (myeloid) versus lymphoid cell types (*p* = 2.8 × 10^−^^3^, one tail *T*-test) but not in the other immune cell types (Fig. 4a). The observation of a high KLF4 activity score in the myeloid cell compartments is both a confirmation of the expectation of KLF4 as a master regulator of myelopoiesis^5^ and a validation of our KLF4 activity score in a controlled scenario. We also noted that, among the TFs associated with tissue remodelling (Fig. 3c, R→R), KLF4 is the TF with the most significant evidence for high activity scores in myeloid relative to lymphoid cells (Table S3).

Next we investigated whether the KLF4 activity score is associated with immune cell infiltration in colorectal cancer samples. Patients were classified as K+ if they had a significant high KLF4 activity score and K− otherwise. Subsequently, we estimated the composition of immune cell types in same cohort of colorectal cancer patients using the computational inference approach CIBERSORT.^9^ This approach estimates the composition of immune cell types in complex tissues using gene expression profile as input.^9^ We observed a significant positive correlation between the KLF4 activity score and the estimated percentage of myeloid cell types: neutrophils, macrophages (M0, M1), mast cells (activated) and dendritic cells (activated) (Fig. 4b, Table S4). In contrast, we observed a significant negative correlation or no correlation between the KLF4 activity and a high percentage of lymphoid cell types: B-cells and T-cells (Fig. 4b). The tail distribution of the estimated myeloid cell fractions is shown in Fig. 4c and d for neutrophils and macrophages (M0), the two myeloid subtypes with higher positive correlation with the KLF4 activity score. The distribution of neutrophils enrichment across K+ patients exhibits a longer tail than for K− patients, independently of the P status. (Fig. 4c, *p* = 3.23 × 10^−^^11^, two-sided Kolmogorov–Smirnov test). Similarly, the distribution of M0 macrophages enrichment across K+ patients also exhibits a longer tail than for K− patients (Fig. 4d, *p* = 5.22 × 10^−^^15^, two-sided Kolmogorov–Smirnov test). These findings support the hypothesis that the odds of having a high KLF4 activity score increase with having a higher composition of neutrophils and M0 macrophages in a tumour sample. In other words, the model suggests that a high KLF4 activity score is a reflection of the myeloid cell infiltration.

## Discussion

We have compared the performance of patient stratification based on an unsupervised clustering (CMS, ref. ^4^) versus the cell proliferation and tissue remodelling reductionism approach (PR, ref. ^3^) in the context of colorectal cancer. Both approaches achieved statistical significances in splitting survival curves. The CMS4 and P−/R+ subtypes have the worst prognosis when using either the unsupervised clustering CMS or the reductionism PR approach, respectively. However, the unsupervised clustering cannot distinguish the rest of the patients in the remaining three subtypes (CMS1,2,3) with respect to survival. In contrast, the classification based on the supervised PR approach is richer. It identified a P+/R− subtype that exhibits significantly better prognosis than P−/R− subtype. In other words, patient classification based on PR contributes an additional prediction that cell proliferation is indicative of good prognosis in colorectal cancer, on top of the current knowledge that tissue remodelling manifests worst prognosis.

At this point we have no clear argument of why increased cell proliferation is an indicator of good prognosis in colorectal cancer. We could speculate that current treatments are better at targeting proliferating cancer cells and, as a consequence, patients harbouring tumours with increased cell proliferation exhibit a better response to therapy. An alternative hypothesis is that increased tissue remodelling is the tumour characteristic causally linked to poor prognosis, while the association of cell proliferation with good prognosis is just a correlation. Since the P and R enrichment scores are negatively correlated, the tumours with low R enrichment scores will generally have high P enrichment scores. However, we should bear in mind that in other cancer types, such as breast and prostate cancer, increased cell proliferation is a marker of poor prognosis.^14–16^ As a matter of fact, there is a dichotomy in the role of cell proliferation in prognosis when looking at different cancer types.^3^ In colorectal and ovarian cancer, increased cell proliferation is indicative of good prognosis but, in brain, breast, lung and prostate cancer it is the other way round.

In the second part of this work, we aimed to identify transcriptional programs that drive the gene expression signatures of PR in colorectal cancer. Among the candidates, we identified KLF4 as a TF whose activity score is significantly correlated with the tissue remodelling enrichment score, which is indicative of poor prognosis in colorectal cancer. We focused on KLF4 because it is one of the reprogramming factors in induced pluripotent stem cells^11, 12^ and it has a documented role in myelopoiesis.^5^ Both of these features could potentially contribute to the malignancy and poor outcome associated with tissue remodeling. We validated the KLF4 signature to be significantly expressed in the myeloid versus lymphoid types of immune cells, in agreement with its role in myelopoiesis.^5^ Furthermore, the KLF4 activity score was found to be positively correlated with the presence of myeloid cells in the colorectal cancer samples. Taken together this analysis indicates that the tissue remodelling signature in colorectal tumours is in part due to the infiltration of myeloid cells and the KLF4 is the TF sustaining the myeloid state in the infiltrating immune cells.

The implication of these observations for the treatment of colorectal cancer with a high degree of tissue remodelling remains to be elucidated. Future work is required to identify what factors drive the enrichment for myeloid types of immune cells in colorectal cancers. A recent study highlighted a role of TGFβ signalling in the exclusion of T-cells (lymphoid lineage) from colorectal tumours in genetically engineered mouse models.^17^ TGFβ is a well known driver of tissue remodelling,^18^ but it is not clear how the exclusion of T-cells influences the abundance of myeloid cell types in colorectal cancers with increased tissue remodelling.

## Electronic supplementary material

Supplementary Tables

Data Set 1

Data Set 2

Data Set 3

Data Set 4

## Availability of data and material

All datasets used in this publication are available from the public sources indicated in the 'Methods' section.
