# Supplementary material for: Analysis of cell proliferation and tissue remodelling uncovers a KLF4 activity score associated with poor prognosis in colorectal cancer
Source: Br J Cancer. 2018 Oct 5;119(7):855–63. doi: 10.1038/s41416-018-0253-0 (PMC6189192; doi:10.1038/s41416-018-0253-0)
Supplement: Supplementary file 1 — Supplementary Tables [file 41416_2018_253_MOESM1_ESM.pdf]

Analysis of cell proliferation and tissue remodelling uncovers a KLF4 activity score associated with poor prognosis in colorectal cancer

Silvia Halim[1], Elke K Markert[2,*] and Alexei Vazquez[1,2,*]

[1]Cancer Research UK Beatson Institute, Glasgow, UK

[2]Institute of Cancer Sciences, University of Glasgow, Glasgow, UK

Supplementary Information

Description

Supplementary Tables 1-4

Table S1: Transcription factors that activate gene expression with correlation of their activity and enrichment of their target genes with P and R signatures. Each correlation or enrichment score is followed by a p-value and an indication if the p-value remains significant after Benjamini-Hochberg (BH) correction.

TF	Spearman Correlation with P	P-value	Significant after BH correction	Spearman Correlation with R	P-value	Significant after BH correction	Enrichment of target with P	P-value	Significant after BH correction	Enrichment of target with R	P-value	Significant after BH correction
PROX1	0.53	1.0E-05	Y	-0.24	1.0E-05	Y	0.17	1.0E-05	Y	0	0.50	N
RUNX3	0.18	1.0E-05	Y	-0.11	2.8E-03	Y	0.12	2.2E-03	Y	-0.13	1.2E-03	Y
SOX2	0.19	1.0E-05	Y	0.06	0.08	N	0.08	0.02	Y	-4.2E-03	0.46	N
TP53	0.09	0.02	Y	-0.36	1.0E-05	Y	0.25	1.0E-05	Y	-7.4E-04	0.49	N
BRCA1	-0.16	6.0E-05	Y	0.60	1.0E-05	Y	0.04	0.17	N	0.34	1.0E-05	Y
CEBPB	-0.02	0.29	N	0.12	1.9E-03	Y	-0.08	0.02	Y	0.44	1.0E-05	Y
CLOCK	-0.26	1.0E-05	Y	0.17	2.0E-05	Y	0	0.50	N	0.39	1.0E-05	Y
CREB1	-0.32	1.0E-05	Y	0.25	1.0E-05	Y	0.08	0.03	N	0.55	1.0E-05	Y
ERG	-0.03	0.24	N	0.26	1.0E-05	Y	0	0.50	N	0.90	1.0E-05	Y
ESR1	-0.40	1.0E-05	Y	0.14	3.0E-04	Y	0.04	0.16	N	0.34	1.0E-05	Y
ETS2	-0.22	1.0E-05	Y	0.27	1.0E-05	Y	0	0.50	N	0.76	1.0E-05	Y
ETV4	-0.41	1.0E-05	Y	0.62	1.0E-05	Y	0	0.50	N	0.66	1.0E-05	Y
FOXO3	-0.29	1.0E-05	Y	0.48	1.0E-05	Y	0	0.50	N	0.27	1.0E-05	Y
GATA3	-0.03	0.24	N	0.32	1.0E-05	Y	-0.21	1.0E-05	Y	0.48	1.0E-05	Y
JUN	-0.20	1.0E-05	Y	0.15	8.0E-05	Y	-0.20	1.0E-05	Y	0.47	1.0E-05	Y
KLF4	-0.09	0.02	Y	0.59	1.0E-05	Y	-0.29	1.0E-05	Y	0.18	1.0E-05	Y
NFE2L2	-0.06	0.09	N	0.32	1.0E-05	Y	0	0.50	N	0.24	1.0E-05	Y
NR5A2	-0.33	1.0E-05	Y	0.21	1.0E-05	Y	0	0.50	N	0.29	1.0E-05	Y
PML	-0.07	0.06	N	0.61	1.0E-05	Y	0	0.50	N	0.30	1.0E-05	Y
PPARD	-0.14	4.9E-04	Y	0.23	1.0E-05	Y	0	0.50	N	0.62	1.0E-05	Y
RELA	-0.11	3.1E-03	Y	0.29	1.0E-05	Y	-0.01	0.41	N	0.43	1.0E-05	Y
SNAI2	-0.09	0.02	Y	0.41	1.0E-05	Y	-0.37	1.0E-05	Y	0.54	1.0E-05	Y
SPI1	-0.03	0.21	N	0.42	1.0E-05	Y	0	0.50	N	0.51	1.0E-05	Y
STAT1	-0.06	0.07	N	0.36	1.0E-05	Y	-0.36	1.0E-05	Y	0.55	1.0E-05	Y

Table S2: Correlation of KLF4 activity with target genes.

KLF4 Target	Correlation with KLF4	P-value	Significant after BH correction
IL1B	0.30	1.0E-05	Y
IL6	0.22	1.0E-05	Y
CCNB1	0.21	1.0E-05	Y
BIRC5	0.19	1.0E-05	Y
GDF15	0.15	1.0E-05	Y
MMP2	0.14	1.0E-05	Y
ODC1	0.13	1.0E-05	Y
KRT19	0.11	1.0E-05	Y
TP53	0.10	1.0E-05	Y
THBD	0.10	1.0E-05	Y
CCND1	0.09	1.0E-05	Y
IFITM3	0.09	1.0E-05	Y
CDH5	0.08	5.0E-05	Y
LAMA3	0.08	7.0E-05	Y
HSPA8	0.08	7.0E-05	Y
ALPI	0.05	5.4E-03	N
BDKRB2	0.05	8.4E-03	N
VDR	0.03	0.06	N
CDKN1A	0.03	0.07	N
RARA	0.02	0.17	N
CDKN1C	0.01	0.29	N
GPA33	0.01	0.37	N
HDC	0.00	0.44	N
ATF3	-0.04	0.02	N
LXN	-0.06	8.4E-04	Y
CDH1	-0.06	7.2E-04	Y
NANOG	-0.07	1.2E-04	Y
CDKN1B	-0.15	1.0E-05	Y

Table S3: Activity scores of putative tissue remodelling TFs in immune cell types.

	T-Test (myeloid vs lymphoid)		
TF	Higher in myeloid	Higher in lymphoid	Two tails
KLF4	2.76E-03	1	5.52E-03
CEBPB	2.83E-03	1	5.65E-03
STAT1	5.99E-03	0.99	0.01
ETS2	0.02	0.98	0.03
ESR1	0.04	0.96	0.08
CLOCK	0.11	0.89	0.22
FOXO3	0.15	0.85	0.29
ETV4	0.17	0.83	0.34
NR5A2	0.17	0.83	0.33
ERG	0.19	0.81	0.38
RELA	0.31	0.69	0.62
NFE2L2	0.32	0.68	0.64
JUN	0.37	0.63	0.73
CREB1	0.41	0.59	0.82
BRCA1	0.83	0.17	0.35
PML	0.86	0.14	0.27
SPI1	0.88	0.12	0.24
PPARD	0.89	0.11	0.23
GATA3	0.99	0.01	0.03
SNAI2	1	6.54E-05	1.31E-04

Table S4: Correlation of KLF4 activity score with inferred immune cell fractions in the colorectal tumour samples.

Immune cell type	Correlation with KLF4	P-value	Significant after BH correction
Macrophages M0	0.23	1.0E-05	Y
Neutrophils	0.22	1.0E-05	Y
Mast cells activated	0.19	1.0E-05	Y
Dendritic cells activated	0.14	1.0E-05	Y
NK cells resting	0.13	1.0E-05	Y
Macrophages M1	0.08	6.0E-05	Y
Dendritic cells resting	0.08	1.0E-04	Y
T cells CD4 memory activated	0.03	0.07	N
B cells memory	0.03	0.11	N
T cells follicular helper	0.02	0.12	N
T cells CD4 naive	0.01	0.31	N
T cells regulatory (Tregs)	0.01	0.32	N
T cells CD8	-0.01	0.24	N
NK cells activated	-0.02	0.20	N
Monocytes	-0.04	0.03	N
Eosinophils	-0.05	0.01	Y
Plasma cells	-0.06	2.6E-03	Y
T cells gamma delta	-0.09	1.0E-05	Y
Macrophages M2	-0.11	1.0E-05	Y
T cells CD4 memory resting	-0.12	1.0E-05	Y
B cells naive	-0.14	1.0E-05	Y
Mast cells resting	-0.16	1.0E-05	Y
